# LOX-1: Regulation, Signaling and Its Role in Atherosclerosis

**DOI:** 10.3390/antiox8070218

**Published:** 2019-07-11

**Authors:** Ajoe John Kattoor, Akshay Goel, Jawahar L. Mehta

**Affiliations:** 1 Division of Cardiology, John H. Stroger, Jr. Hospital of Cook County, Chicago, IL 60612, USA; 2 Division of Cardiology, Central Arkansas Veterans Healthcare System and the University of Arkansas for Medical Sciences, Little Rock, AR 72205, USA

**Keywords:** LOX-1, ox-LDL, atherogenesis, atherosclerosis, oxidative stress

## Abstract

Atherosclerosis has long been known to be a chronic inflammatory disease. In addition, there is intense oxidative stress in atherosclerosis resulting from an imbalance between the excess reactive oxygen species (ROS) generation and inadequate anti-oxidant defense forces. The excess of the oxidative forces results in the conversion of low-density lipoproteins (LDL) to oxidized LDL (ox-LDL), which is highly atherogenic. The sub-endothelial deposition of ox-LDL, formation of foamy macrophages, vascular smooth muscle cell (VSMC) proliferation and migration, and deposition of collagen are central pathophysiologic steps in the formation of atherosclerotic plaque. Ox-LDL exerts its action through several different scavenger receptors, the most important of which is LOX-1 in atherogenesis. LOX-1 is a transmembrane glycoprotein that binds to and internalizes ox-LDL. This interaction results in variable downstream effects based on the cell type. In endothelial cells, there is an increased expression of cellular adhesion molecules, resulting in the increased attachment and migration of inflammatory cells to intima, followed by their differentiation into macrophages. There is also a worsening endothelial dysfunction due to the increased production of vasoconstrictors, increased ROS, and depletion of endothelial nitric oxide (NO). In the macrophages and VSMCs, ox-LDL causes further upregulation of the LOX-1 gene, modulation of calpains, macrophage migration, VSMC proliferation and foam cell formation. Soluble LOX-1 (sLOX-1), a fragment of the main LOX-1 molecule, is being investigated as a diagnostic marker because it has been shown to be present in increased quantities in patients with hypertension, diabetes, metabolic syndrome and coronary artery disease. LOX-1 gene deletion in mice and anti-LOX-1 therapy has been shown to decrease inflammation, oxidative stress and atherosclerosis. LOX-1 deletion also results in damage from ischemia, making LOX-1 a promising target of therapy for atherosclerosis and related disorders. In this article we focus on the different mechanisms for regulation, signaling and the various effects of LOX-1 in contributing to atherosclerosis.

## 1. Introduction

Atherosclerosis is a chronic inflammatory disease and is characterized by lipid and inflammatory cell deposits in the walls of medium and large sized arteries. There is an interplay between the generation of reactive oxygen species (ROS) and the anti-oxidant defense system, the imbalance of which leads to increased oxidative stress contributing to endothelial dysfunction, which is a major determinant of atherogenesis. In conditions such as hypertension, diabetes, smoking and dyslipidemia, which are known to elevate the cardiovascular disease risk, ROS production has been found to be increased in the vessel walls [1]. Increased ROS species leads to the oxidation of native LDL to oxidized LDL (ox-LDL), which plays a significant role in atherogenesis. 

Ox-LDL is defined as “circulating LDL derived particle that have peroxides or their degradation products within it or associated with the particle” [2]. Ox-LDL exerts its various effects on cells such as endothelial cells, platelets, macrophages, fibroblasts, and smooth muscle cells through the transmembrane glycoprotein LOX-1 [3]. In addition, other scavenger receptors (SR), such as CD-36 and SR-A, also contribute to the internalization of ox-LDL and atheroma formation.

In this chapter, we will discuss the role of LOX-1 in contributing to atherogenesis and its major complication—myocardial ischemia.

## 2. Mechanism of Atherosclerosis

The formation of fatty streaks is considered the initial step in atherogenesis. Fatty streaks are subendothelial deposits of lipid-laden macrophages. Endothelial dysfunction is the primary step in fatty streak formation. In classic “risk factors” for atherosclerosis, such as smoking, hypertension, diabetes and dyslipidemia, there is an activation of endothelial cells resulting in adhesion molecules expression which facilitates the attachment of inflammatory cells primarily circulating monocytes. These cells migrate into the sub-endothelial region in response to chemotactic signals. Various growth factors stimulate the expression of various SRs and the differentiation of monocytes into macrophages that internalize the modified lipids resulting in the formation of foam cells—the hallmark of early atherosclerotic lesion. 

The nascent atheroma then develops under the influence of proliferating and migrating smooth muscle cells and the deposition of an extracellular matrix in response to various growth factors released by macrophages to form advanced atherosclerotic plaque [1]. The local inflammatory reaction causes the release of matrix metalloproteinases (MMPs), which leads to the loss of endothelium due to the degradation of the subendothelial basement membrane, which in turn causes plaque disruption and thrombus formation, as well as the sudden expansion of the plaque. Often, anticoagulant pathways override this and a healing process begins. Due to the release of the platelet derived growth factor (PDGF) and tissue growth factor-β (TGF-β), there is smooth muscle migration, proliferation and deposition of collagen, forming a fibrous cap for the atherosclerotic plaque. The fibrous cap is weakened by MMPs and can lead to a plaque rupture, leading to the exposure of the thrombogenic tissue factor in the lipid core to the coagulation factors in the cells, leading to thrombosis in the blood vessel [4]. 

## 3. Ox-LDL–LOX-1 in Atherogenesis

Ox-LDL plays an important role in atherogenesis. It inhibits the constitutive endothelial nitric oxide synthetase (eNOS) expression and contributes to the generation of ROS from cells, smooth muscle cells and macrophages [4]. It causes the induction of the expression of adhesion molecules on endothelial cells, macrophage proliferation, collagen formation, smooth muscle cell migration and platelet activation. Multiple SRs have been identified based on their role in scavenging modified lipids. They have been classified into 8 classes, namely classes A through H. They bind to various ligands, including modified self-proteins and pathogenic organisms. All SRs bind to modified LDLs, except SCARA-5 (class A) and LAMP (Class D) [5]. SR-A type 1 and II, CD-36 and LOX-1 have been found to be involved in the formation of foam cells by the uptake of modified LDL [6]. In vitro studies suggest that SR-A and CD36 contribute to up to 75% to 90% of the ox-LDL uptake [7]. SR-As are normally expressed in myeloid cells, but in the presence of oxidative stress and growth factors, they are expressed in endothelial cells and smooth muscle cells [8]. The deletion of SR-A has been shown to decrease the size of atherosclerotic lesions in Apo-E null mice. In addition, there is an approximately 60% reduction in the uptake of modified LDL in macrophages of SR-AI and SR-AIII deficient mice [9]. SR-A also mediates the adhesion of macrophages to the extracellular matrix [10]. CD36 is found on monocytes, macrophages, platelets and adipocytes [5]. CD36 contribute to foam cell formation by macrophages by mediating the binding and uptake of modified lipids. Human macrophages lacking CD36 have a 40% decrease in the binding and uptake of ox-LDL [11]. CD36 also mediates the adhesion between the macrophages and activated platelets and collagen.

Among SRs, LOX-1, a member of the class E SR family, plays a critical role in atherosclerosis. LOX-1 is a 50-kDA transmembrane glycoprotein that was initially identified in bovine aortic endothelial cells, where it acted as an important receptor for the binding, internalization and degradation of ox-LDL [3]. It has a cytoplasmic, transmembrane neck and extracellular domains and was later found to be expressed in a variety of other cells, such as macrophages, vascular smooth muscle cells, cardiomyocytes, platelets and fibroblasts. 

LOX-1, in addition to the binding and internalization of ox-LDL, contributes to endothelial dysfunction and apoptosis and helps in the formation of foam cells in macrophages and vascular smooth muscle cells. Mediators such as angiotensin II, cytokines, sheer stress, and advanced glycation end-products, and conditions such as diabetes mellitus, hypertension and dyslipidemia, upregulate LOX-1 [12,13,14]. In addition to ox-LDL, several other molecules act as ligands to the LOX-1 receptor. Tumor necrosis factor-alpha (TNF-α), interleukin-1 (IL-1), interferon-gamma (IFN-γ), and modified lipoproteins such as glycoxidized LDL, lysophosphatidylcholine and ROS, induce the expression of LOX-1. In in-vivo studies, hypertension and diabetes, obesity, ischemia reperfusion injury, heart failure, psychological stress and HIV infection have been shown to increase the LOX-1 expression [15,16]. 

## 4. Regulation of LOX-1

The human LOX-1 protein is encoded by the ox-LDL receptor 1 gene (*OLR1*) in the C-type lectin gene cluster of chromosome 12. NF-κB and AP-1 binding elements are present in the 5’ -regulatory regions of the LOX-1 gene [17]. The extracellular domain of the LOX-1 protein, when cleaved from the neck region, forms the soluble LOX-1 (sLOX-1). LOX-1 expression is low under normal physiological conditions. However, in the presence of an inflammatory milieu that is rich in the above-mentioned ligands, there is an upregulation of the transcription and translation of the LOX-1 gene [16]. Ox-LDL is the most potent activator of LOX-1. The binding of ox-LDL to LOX-1 activates NF-κB (nuclear factor kappa-light-chain-enhancer of activated B cells). NF-κB binds at the 5’ side of LOX-1 to the shear stress responsive element binding site GAGACC and activates the expression of LOX-1. The binding of ox-LDL to LOX-1 results in an increased expression of adhesion molecules like vascular cell adhesion molecule-1 (VCAM-1), and cytokines including monocyte-chemoattractant-protein-1 (MCP-1) [17]. These pro-inflammatory molecules themselves in turn cause the increased expression of LOX-1 in endothelial cells, resulting in a self-perpetuating cycle between ox-LDL, LOX-1 and NF-κB [18]. Certain other pro-inflammatory and pro-atherogenic molecules like IL-1 and-6 and TNF-α upregulate the LOX-1 expression in vascular smooth muscle cells. In addition, the LOX-1 activation worsens oxidative stress, resulting in more ox-LDL formation, and this cycle self-amplifies [16].

The exposure of endothelial cells to homocysteine (such as in hyperhomocysteinemia) has been shown to increase LOX-1 mRNA transcription and translation, which is reversed with folic acid [19,20]. Certain infectious agents like *Chlamydia*, *H. pylori* and cytomegalovirus, which have been implicated in atherosclerosis, increase sLOX-1 levels and LOX-1 directed ox-LDL endocytosis in human umbilical vein endothelial cells [21].

Besides inflammation and atherosclerosis, LOX-1 expression is regulated by several epigenetic mechanisms [22]. MicroRNAs (miRNAs) are non-coding RNAs that are responsible for post-transcription gene expression modulation. Several studies have shown that various miRNAs like miR-155, miR-590-5P and let-7 g regulate LOX-1 expression, mainly in a reciprocal fashion. For instance, the knockdown or silencing of miR-155 upregulates the LOX-1 and ox-LDL mediated lipid uptake. It also increases the production of IL-1 and -8 and TNF-α [23]. On the other hand, miR-590-5p binds to the 3’ untranslated region of LOX-1 and degrades it. As expected, treatment with miR-590-p mimicking drugs suppresses LOX-1 and attenuates angiogenesis in human umbilical vein endothelial cells [24,25]. When apoE knockout mice were given a high fat diet, there was an upregulation of LOX-1 in the aorta, leading to increased foam cells and lipid deposition. This upregulation of LOX-1 and associated vessel wall pathology changes were inhibited by a pre-treatment with let-7 g agonists [26].

DNA methylation suppresses transcription and is another epigenetic mechanism responsible for the modulation of the LOX-1 expression. Ox-LDL downregulates the DNA methylation of the LOX-1 promoter, thereby increasing the production of LOX-1 [27]. Analogously, the exposure of endothelial cells to homocysteine decreased DNA methyltransferase activity and increased LOX-1 expression [28]. These studies suggest that atherogenic stimuli have the potential to increase LOX-1 levels by repressing DNA methylation [22].

There is also some data on the effect of histone acetylation-deacetylation on the LOX-1 gene regulation. Ox-LDL can worsen inflammation through histone acetylation and the resulting upregulation of the IL-8 and MCP-1 production [29]. In contrast, sirtuin 1 causes the histone deacetylation of NF-κB and downregulates the LOX-1 expression [30]. 

In a recent study, Mentrup et al. showed that LOX-1 signaling is also controlled by intramembrane proteases such as signal peptide peptidase-like 2a and b (SPPL2a/b) that are involved in regulated intramembrane proteolysis [31]. Regulated intramembrane proteolysis involves a sequential cleavage of the substrate ectodomain of the transmembrane protein followed by the processing of the residual membrane-embedded stub by the intramembrane cleaving proteases (I-CLIP). The resulting intracellular domain of the transmembrane protein is released into the cytosol and can contribute to various regulatory functions [32]. SPPL2a/b are I-CLIPs that cleave N-terminal fragments (NTFs) derived from the regulated intramembrane proteolysis of transmembrane proteins. LOX-1 was found to be processed by ADAM10 and lysosomal proteases to form its NTF. LOX-1 NTFs were found to activate MAP kinases independent of LOX-1 ligands. They triggered downstream signaling that induced proatherogenic and fibrotic targets. The LOX-1 NTFs undergo intramembrane cleavage by SPPL2a/b. Unlike the ligand-induced full length receptor, the NF-κB pathway is not activated by the LOX-1 NTF induced signaling. The inhibition of SPPL2a/b in double deficient mice caused increased ligand dependent LOX-1 induced MAPK signaling when challenged with hypercholesterolemia. Thus, the Mentrup et al. study suggests the presence of two different LOX-1 signaling pathways [31].

Stancel et al. have proposed that C-reactive protein (CRP) and LOX-1 form a cyclic mechanism with ox-LDL in atherogenesis [33]. CRP is a ligand for LOX-1 and increases vascular permeability, impairs the endothelium-dependent vasodilator function and plays a role in monocyte-endothelial cell adhesion. CRP, through FcγRI/CD64 and FcγRIIa/CD32, increased the expression of LOX-1 [34]. Also, oxLDL and plasma L5, through a LOX-1 dependent manner, were found to increase CRP release from human aortic endothelial cells [35].

## 5. LOX-1 Signaling Pathways and Its Effects

LOX-1 activation results in several downstream signaling pathways. LOX-1 binds to MMP14 and activates RhoA and Rac1. RhoA inhibits the endothelial NO synthesis, while Rac1 increases the NADPH oxidase activity, resulting in ROS production and oxidative stress. Hence, the inhibition of LOX-1 binding to MMP14 will reduce oxidative stress [36]. p66^shc^ is a redox enzyme involved in mitochondrial ROS production and mitochondrial DNA damage. The exposure of endothelial cells to a high ox-LDL level phosphorylates p66^shc^. This causes more mitochondrial DNA damage and increases plaque formation and instability. Treatment with siRNA has a protective effect on mitochondria [37,38,39]. NADPH oxidase (Nox) enzymes are a major source of ROS in the vascular tissues. The Nox2 isoform is mainly located in monocytes and forms an active complex with the subunits p22^phox^, p47^phox^, p40^phox^, and p67^phox^ [40]. On the other hand, Nox4 is expressed in endothelial cells and forms a complex with p22^phox^ [41,42]. The Ox-LDL induced ROS formation is contributed by the induction of Nox,2, Nox4 and p47^phox^ [43,44]. The deletion of LOX-1 in mice fed with high cholesterol and a high fat diet decreased the expression of Nox2, Nox4, p22^phox^, and p47^phox^ [45,46]. NF-κB is activated by ox-LDL through LOX-1. This activates the proinflammatory pathways and LOX-1, thus forming a vicious cycle of increased ox-LDL uptake through the activated LOX-1, increased ROS formation and increased expression of the LOX-1 receptors [16]. 

The interaction between ox-LDL and LOX-1 results in an increased production of adhesion molecules like VCAM-1 and cytokines like MCP-1, leading to the attachment of monocytes to endothelial cells. Ox-LDL–LOX-1 binding leads to the phosphorylation of mitogen-activated protein kinase (MAPK) and induces apoptosis [43]. Ox-LDL causes protein kinase C activation and plays a critical role in the expression of MMPs [47]. LOX-1 induction also reduces the phosphorylation of protein kinase B (PKB), which is involved in the activation of eNOS by its phosphorylation. Thus, the decrease in PKB phosphorylation decreases the NO production due to the decline in the eNOS activation [48,49]. 

The increased proprotein convertase subtilsin/kexin type 9 (PCSK9) expression upregulates the LOX-1-mediated ox-LDL uptake [50]. LPS increases the LOX-1 expression and PCSK9 levels in human endothelial cells and vascular smooth muscle cells. The data from gene knockout mice and siRNA transfection studies have shown that LOX-1 and PCSK9 upregulate each other’s gene expression [51].

The mTOR inhibitor rapamycin downregulates LOX-1 by interfering with the signaling interactions between mTOR, LOX-1 and NF-κB. Rapamycin and LOX-1 inhibition is associated with decreased autophagy, implying the role of LOX-1 in regulating autophagy [52,53]. In addition, LOX-1 is involved in the activation of the NOD-like receptor pyrin domain containing (NLRP3) inflammasome and in the increased expression of the angiotensin converting enzyme (ACE) [40].

**Figure 1:**
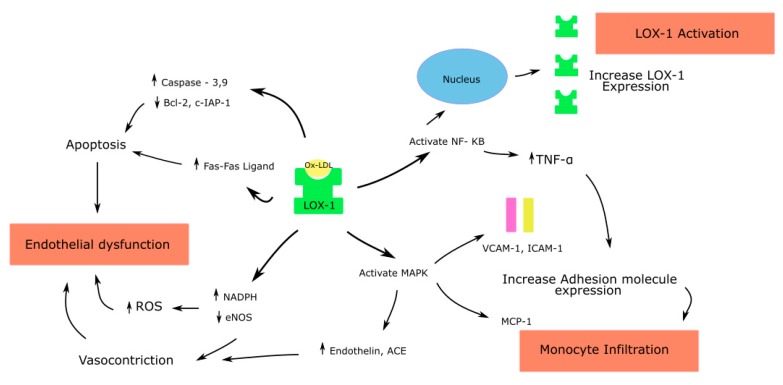
Effects of Ox-LDL–LOX-1 interaction in endothelial cells; ROS—Reactive oxygen species; endothelial nitric oxide synthase—eNOS; Nuclear factor kappa-light-chain-enhancer of activated B cells—NF-κB; Mitogen activated protein kinase—MAPK; Angiotensin converting enzyme—ACE; Monocyte chemoattractant protein—MCP; Tumor necrosis factor—TNF.

**Figure 2:**
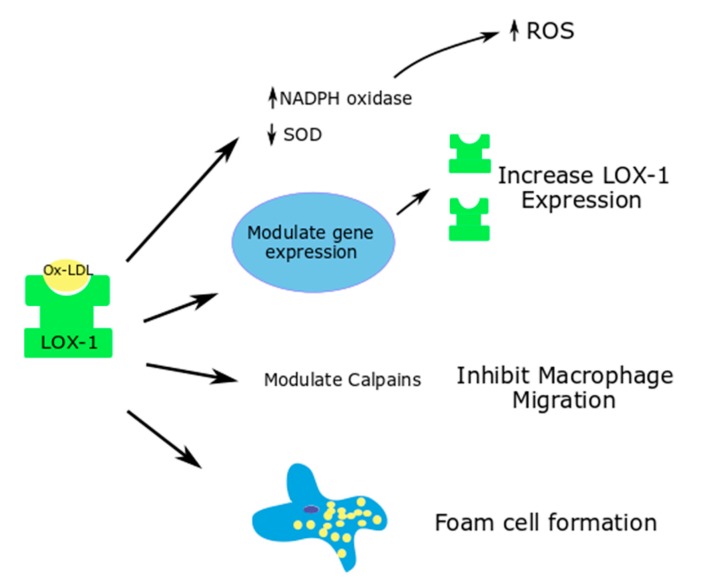
Effects of ox-LDL-LOX-1 interaction in macrophages; Superoxide dismutase—SOD.

**Figure 3:**
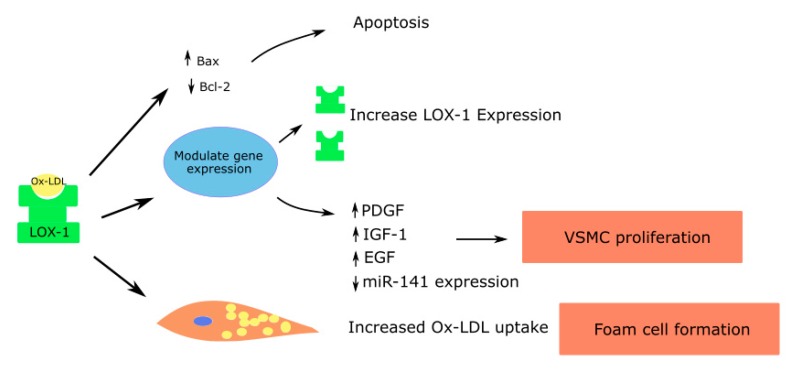
Role of LOX-1 in vascular smooth muscle cells contributing to atherosclerosis; Platelet derived growth factor—PDGF; Insulin like growth factor—IGF; Epidermal growth factor—EGF.

Ox-LDL binding to LOX-1 causes the rapid internalization of the ligand receptor complex into the cell. Based on the type of cell where this interaction occurs, downstream signaling leads to varied effects, as described below (Figure 1, Figure 2 and Figure 3). 

### 5.1. Endothelial Cells

Through LOX-1, ox-LDL causes endothelial dysfunction by multiple pathways [15,54] (Figure 1). The ox-LDL–LOX-1 pathway activates the mitogen-activated protein kinase (MAPK), causing an increase in the MCP-1 expression and monocyte adhesion. Human coronary endothelial cells (HCAECs), when incubated in the presence of antisense oligonucleotides to the 5’ coding sequence of the *LOX-1* gene, suppressed the basal LOX-1 protein and LOX-1-mRNA. It also significantly decreased the ox-LDL mediated upregulation of MCP-1 and monocyte adhesion [55]. These, in addition to the increased expression of cell adhesion molecules such as VCAM-1 and ICAM-1, promote the migration and differentiation of the monocytes to macrophages, a critical step in atherogenesis [55]. 

Ox-LDL increases the generation of vasoconstrictors such as the angiotensin converting enzyme (ACE) and endothelin-1 [47,56]. Cultured HCAECs, when incubated with ox-LDL, showed an increased expression of ACE in a time- and concentration-dependent manner. Pretreatment with an antibody to LOX-1 was able to block this action of ox-LDL. Native-LDL had no major effect on the ACE expression in these cells. This effect of ox-LDL is likely mediated through the MAPK activation, as the pretreatment of HCAECs with the MAPK p42/44 inhibitor attenuated the ACE expression [47]. 

Ox-LDL also increases ROS by increasing the NADPH oxidase activity. This causes the inactivation of NO. In addition, ox-LDL causes the dysfunction of the eNOS enzyme by displacing it from the caveolar membrane location [57] and by arginase II activation, which in turn downregulates eNOS due to competition for its common substrate L-arginine [58]. Thus, the increased generation of vasoconstrictors and ROS, and the depletion of NO, lead to endothelial dysfunction. 

Ox-LDL activates caspase-3 and caspase-9 in the intrinsic apoptotic pathway and inhibits antiapoptotic proteins such as B-cell lymphoma 2 (Bcl-2) and the cellular inhibitor of apoptosis protein1 (c-IAP-2) [59]. Chen et al., in HCAECs treated with ox-LDL, showed a time- and concentration-dependent increase in the release of activators of caspase-9, cytochrome c and Smac from mitochondria to cytoplasm and the activation of caspase-9 [59]. Ox-LDL was shown to upregulate Fas expression in the extrinsic apoptotic pathway on the endothelial cell surface, thereby leading to Fas mediated apoptosis [60]. Thus, Ox-LDL promotes apoptosis of endothelial cells by both intrinsic and extrinsic pathways.

Ox-LDL activates NF-κB, and its activation in turn increases the expression of TNF-α, adhesion molecules and LOX-1 in endothelial cells. The LOX-1 gene has a NF-κB binding site at its 5’ flanking region, and therefore ox-LDL increases the expression of LOX-1 through NF-κB, leading to a vicious cycle of proinflammatory signaling [17]. 

Ox-LDL also increases the synthesis of MMP-1, MMP-3 and MMP-9, causing an imbalance in the MMPs in endothelial cells, contributing to the increased degradation of fibrotic cap and predisposition to atherosclerotic plaque rupture [61,62]. 

### 5.2. Macrophages

In normal circumstances, LOX-1 expressed in macrophages contributes to 5–10% of the ox-LDL uptake. However, in proinflammatory states, the expression of LOX-1 is upregulated, and it contributes to around 40% of the ox-LDL update by macrophages [15,63]. Proinflammatory cytokines and hyperglycemia upregulate this phenomenon and increase the uptake of ox-LDL, thereby contributing to lipid accumulation and foam cell formation [64]. 

Through LOX-1, ox-LDL modulate cell-dependent proteases such as calpains, which are important in macrophage migration (Figure 2). Ox-LDL causes an increased macrophage attachment, increases the calcium concentration in the cells and inhibits macrophage migration. These functions of ox-LDL in macrophages were shown to be reversed with LOX-1 deletion [65]. 

Yang et al. used siRNA technology to study the effects of LOX-1 silencing on the ox-LDL induced ROS generation and Nox expression in mouse macrophages. Ox-LDL caused an increase in the ROS generation and a decrease in the superoxide dismutase activity in the macrophages at a cellular level, thus contributing to apoptosis. LOX-1 siRNA was able to significantly reverse the oxidative stress parameters [64]. 

### 5.3. Smooth Muscle Cells

Like endothelial cells and macrophages, LOX-1 is expressed on the cell membranes of vascular smooth muscle cells, and is upregulated in inflammatory states in response to cytokines that are proinflammatory such as TNF-α, IL-1 and IFN-γ [66].

Micro RNA let-7g, which inhibits the LOX-1 gene, was shown to reduce ox-LDL mediated vascular smooth muscle cell migration and proliferation [67]. Ox-LDL induces the release of growth factors such as the insulin-like growth factor (IGF-1), PDGF and epidermal growth factor (EGF), leading to vascular smooth muscle cell proliferation [4,66,68]. In addition, ox-LDL suppresses the miR-141 expression, which in turn promotes the proliferation of vascular smooth muscle cells [69] (Figure 3). 

Vascular smooth muscle cells can also form foam cells through the accumulation of lipid droplets. The anti-LOX-1 antibody can significantly decrease the uptake of ox-LDL and formation of foam cells by vascular smooth muscle cells [70]. Elevated concentrations of ox-LDL increase the proapoptotic protein expression such as the bcl-2-associated X protein (Bax), causing apoptosis of vascular smooth muscle cells. Apoptosis in vascular smooth muscle cells leads to atherosclerotic plaque instability and rupture. The effects are mediated largely through LOX-1 [71].

### 5.4. Platelets and Fibroblasts

LOX-1 expression on the platelets occurs in an activation-dependent manner unlike scavenger receptors such as CD36, which are constitutively expressed. In activated platelets, LOX-1 mediates the binding and internalization of ox-LDL [72]. The activated platelets induce the expression of platelet adhesion molecules, induce foam cell formation and contribute to endothelial dysfunction [73,74]. LOX-1 is important in thrombus formation by contributing to the ADP-induced activation of fibrinogen receptors such as alpha (IIb)beta(3) and alpha(2)beta(1) integrins. The anti-LOX-1 antibody was shown to inhibit the ADP-induced platelet aggregation [75]. Chan et al. examined the effect of L5 (highly electronegative LDL) in thrombus formation and demonstrated that L5 enhanced the ADP-induced platelet activation and was contributed by the LOX-1 mediated Protein Kinase C signaling pathway [76]. Aspirin and statins have also been shown to reduce the expression of LOX-1 in platelets. Activated platelets also contribute to endothelial dysfunction by inducing endothelin-1 in endothelial cells, through its interaction with LOX-1 and CD40 [77]. In addition, through LOX-1, ox-LDL can cause plaque instability through the release of the extracellular MMP inducer CD147 [78]. 

In fibroblasts, the anti-LOX-1 antibody was shown to decrease the effects of TGF-β mediated collagen synthesis [79]. In addition, the LOX-1 deletion in LDL receptor knockout mice decreased collagen accumulation in plaque [45]. Thus, LOX-1 expression in fibroblast contributes to collagen formation.

## 6. LOX-1 as a Diagnostic Marker and Therapeutic Target

The extracellular portion of the LOX-1 receptor can get cleaved by the action of ADAM10 metalloproteinase and form sLOX-1. The sLOX-1 measurement is being investigated as a diagnostic marker for various cardiovascular diseases. There are reports that sLOX-1 can be used as an early predictor for endothelial dysfunction in metabolic syndrome [80]. Elevated sLOX-1 levels have been associated with hypertension, diabetes mellitus type 2 and smoking in various studies; however, the interpretation of these results is difficult as these risk factors often coexist in patients with cardiovascular diseases [81,82,83]. 

There have been reports of a correlation between sLOX-1 receptor levels in patients with acute coronary syndrome. In a study by Hayashida et al., the sLOX-1 levels were significantly higher in acute coronary syndrome patients (*n* = 521) and showed an earlier peak than troponin T. This suggested that sLOX-1 may be considered an early marker of plaque instability [84].

The levels of sLOX-1 were found to be higher in the coronary circulation than in the systemic circulation in patients with acute coronary syndrome and exertional angina, suggesting its origin from the coronary circulation [85]. sLOX-1 levels were also studied in percutaneous coronary intervention related peri procedural myocardial infarction (PCI-RPMI) patients and was found to be higher in those undergoing PCI for stable angina who subsequently developed PCI-RPMI. Thus, the use of sLOX-1 may be helpful in evaluating coronary events in this patient group [86]. 

We believe that large-scale studies are needed for the application of sLOX-1 levels in diagnosing cardiovascular diseases in clinical practice.

## 7. LOX-1 Directed Therapy in Atherosclerosis and Myocardial Ischemia

Evidence of the benefits of eliminating LOX-1 came from the study by Hinagata et al. in a rat model of neointimal hyperplasia after a balloon arterial injury [87]. Mehta et al. [88] showed that the LOX-1 gene deletion was associated with a decrease in oxidative stress, inflammatory response, NO degradation and atherosclerosis in LDL-receptor null mice that were on a high cholesterol diet for 18 weeks. In another study by Hu et al., the LOX-1 deletion in LDL-receptor null mice decreased collagen formation in atherosclerotic regions [45].

Besides atherosclerosis, studies have shown the benefit of anti-LOX-1 therapy in myocardial ischemia. LOX-1 levels are upregulated in the heart following a short duration of coronary artery occlusion followed by reperfusion. In a study by Li et al. [89], a prior treatment of rats with a LOX-1 antibody was associated with decreased inflammation, apoptosis and myocardial infarct size. Lu et al. [90] showed that LOX-1 gene deletion reduced myocardial injury, and improved the cardiac function and overall survival in mice who underwent permanent coronary artery ligation.

## 8. LOX-1 Inhibitors

Being a contributor in multiple pathways in atherogenesis and related diseases, LOX-1 is considered a potential therapeutic target. Many of the current therapies such as aspirin, statin and oral hypoglycemics exert indirect effects on the LOX-1 expression [91,92]. Many naturally occurring compounds and commonly used herbal drugs modify the LOX-1 expression and affect various steps in atherosclerosis. Gingko biloba extract, curcumin, bergamot peet, and ellagic acid have been shown to decrease the LOX-1 expression. Resveratrol, tanshionone II-A and berberine decrease the ox-LDL mediated ROS generation and thereby decrease atherosclerosis [93]. Quercetin has an antioxidant activity and inactivates the STAT3 signaling pathway, leading to the inhibition of the ox-LDL- and LPS-induced LOX-1 expression and lipid accumulation in macrophages [94]. The active compound in danshen, a medicinal herb used in various cardiovascular diseases, namely dihydrotanshinone I, has been shown to decrease the LPS-induced LOX-1 expression and oxidative stress in human umbilical vein endothelial cells. In-vivo studies using ApoE knockout mice fed with dihydrotanshinone I also showed a decline in the LOX-1 expression, oxidative stress and atherosclerotic plaque formation [95]. In a recent study, Yan et al. showed that Longhu Rendan, a traditional Chinese medicine, was able to decrease the LOX-1 expression and levels of cholesterol, LDL-c and triglyceride in apolipoprotein E gene knockout mice [96].

Currently synthetic LOX-1 modulators are being developed based on RNA interference techniques, structure-based drug designs and the use of monoclonal antibodies. In the structure of LOX-1, there is a hydrophobic tunnel that acts as the primary binding site for the phospholipid moiety of ox-LDL. Molecules that bind in this tunnel prevent the interaction of ox-LDL and LOX-1 and are currently being studied. One such molecule developed by Falconi et al., PLAzPC, markedly reduced the interaction between ox-LDL and LOX-1 [97]. By virtual screening techniques, molecular structure databases have been analyzed, and 5 molecules were selected by Thakkar et al. that could potentially inhibit LOX-1. Among them, two of the molecules were shown to significantly inhibit the downstream signaling, LOX-1 mRNA expression and ox-LDL uptake in endothelial cells [98]. MiRNAs are noncoding RNAs that modify the gene expression by exerting post-transcription effects. Let7g miRNA has been used to inhibit the LOX-1 expression and ox-LDL uptake in human aortic smooth muscle cells [99]. Studies using small interfering RNAs, such as antisense *OLR1*, showed the downregulation of LOX-1 mRNA and protein expression using the RNA interference technique. The development of monoclonal antibodies against LOX-1 is challenging due to the highly conserved C-type lectin domain of LOX-1 among mammalian species [93]. Studies with chimeric chicken-human antibodies have demonstrated the ability to inhibit the LOX-1 effects and decrease the ox-LDL uptake. These antibodies were developed after immunizing chicken with recombinant human LOX-1. Further studies to develop these chimeric molecules, which can be put to clinical use, are being performed.

LOX-1 has also been implicated in collagen deposition leading to scar formation and remodeling in the ischemic heart. LOX-1 gene deletion has been shown to reduce cardiac remodeling signals, resulting in a preserved cardiac contractility after a sustained myocardial ischemia [90].

## 9. Conclusions

Oxidized LDL through LOX-1 plays a critical role in atherogenesis. It influences multiple cell types such as endothelial cells, VSMCs, fibroblasts, macrophages and platelets in the atherosclerotic pathway contributing to endothelial dysfunction, apoptosis, monocyte migration and macrophage differentiation, smooth muscle proliferation and migration and plaque instability—some of the critical steps in atherosclerosis. The sLOX-1 receptor is currently being studied as a potential biomarker for cardiovascular diseases. Furthermore, studies are being conducted that evaluate the use of LOX-1 as a therapeutic target to modify atherosclerosis and related diseases.
